# Genomic analyses of new genes and their phenotypic effects reveal rapid evolution of essential functions in *Drosophila* development

**DOI:** 10.1371/journal.pgen.1009654

**Published:** 2021-07-09

**Authors:** Shengqian Xia, Nicholas W. VanKuren, Chunyan Chen, Li Zhang, Clause Kemkemer, Yi Shao, Hangxing Jia, UnJin Lee, Alexander S. Advani, Andrea Gschwend, Maria D. Vibranovski, Sidi Chen, Yong E. Zhang, Manyuan Long

**Affiliations:** 1 Department of Ecology and Evolution, The University of Chicago, Chicago, Illinois, United States of America; 2 State Key Laboratory of Integrated Management of Pest Insects and Rodents & Key Laboratory of Zoological Systematics and Evolution, Institute of Zoology, Chinese Academy of Sciences, Beijing, China; 3 University of Chinese Academy of Sciences, Beijing, China; 4 Department of Horticulture & Crop Science, The Ohio State University, Columbus, Ohio, United States of America; 5 Department of Genetics and Evolutionary Biology, University of São Paulo, São Paulo, Brazil; 6 Department of Genetics, Yale School of Medicine, West Haven, Connecticut, United States of America; 7 Center for Excellence in Animal Evolution and Genetics, Chinese Academy of Sciences, Kunming, Yunnan, China; University of Münster, GERMANY

## Abstract

It is a conventionally held dogma that the genetic basis underlying development is conserved in a long evolutionary time scale. Ample experiments based on mutational, biochemical, functional, and complementary knockdown/knockout approaches have revealed the unexpectedly important role of recently evolved new genes in the development of *Drosophila*. The recent progress in the genome-wide experimental testing of gene effects and improvements in the computational identification of new genes (< 40 million years ago, Mya) open the door to investigate the evolution of gene essentiality with a phylogenetically high resolution. These advancements also raised interesting issues in techniques and concepts related to phenotypic effect analyses of genes, particularly of those that recently originated. Here we reported our analyses of these issues, including reproducibility and efficiency of knockdown experiment and difference between RNAi libraries in the knockdown efficiency and testing of phenotypic effects. We further analyzed a large data from knockdowns of 11,354 genes (~75% of the *Drosophila melanogaster* total genes), including 702 new genes (~66% of the species total new genes that aged < 40 Mya), revealing a similarly high proportion (~32.2%) of essential genes that originated in various *Sophophora* subgenus lineages and distant ancestors beyond the *Drosophila* genus. The transcriptional compensation effect from CRISPR knockout were detected for highly similar duplicate copies. Knockout of a few young genes detected analogous essentiality in various functions in development. Taken together, our experimental and computational analyses provide valuable data for detection of phenotypic effects of genes in general and further strong evidence for the concept that new genes in *Drosophila* quickly evolved essential functions in viability during development.

## Author summary

It is conventionally believed that the genes in control of important biological functions, processes and structures are conserved in evolution. Using recently developed RNAi knockdown libraries in combination of CRISPR knockout analyses, we showed that a high proportion in a large number of evolutionary new genes (~32.2% in 702 new genes, aged < 40 million years ago) in *D*. *melanogaster* are essential for the survival in development. We found that the frequency of essentiality in gene functions is stable in the species’ various ancestral stages and unchanged among ancient genes, unveiling a constant gene evolution that has driven evolution of *Drosophila* species in the *Sophophora* subgenus. We detected the transcriptional compensation effect from CRISPR knockout for highly similar duplicate copies. We experimentally examined the reproducibility, knockdown efficiency and performance of knockdown libraries, revealing the validness of RNAi knockdown in detection of essential phenotypic effects of genes. Our experimental and computational analyses provided strong evidence for the concept that evolutionary new genes in *Drosophila* quickly evolved essential functions.

## Introduction

The question of how often evolutionary new genes develop essential functions is a critical problem in understanding the genetic basis of development and general phenotypic evolution. New genes in evolution have widely attracted discussion [1–6], supported by increasing studies with fulsome evidence in various organisms [7–13]. The detected large number of new genes with unexpected rate of new gene evolution [14–16] and the revealed important functions of new genes [2,17–20] challenged a widely held dogma that the genetic basis in control of development is conserved in a long time scale of evolution [21–24].

Our previous genome-wide analysis used the RNAi knockdown in a smaller sample showing that new genes may quickly become essential in *Drosophila* and that potential for a gene to develop an essential function is independent of its age [25]. This work suggests a tremendous and quickly evolving genetic diversity, which had not been previously anticipated. Since then, genomes of better quality from more species have allowed for more reliable new gene annotation [15]. In addition, technical progress in the detection of gene effects has increased with better equipped knockdown libraries and direct CRISPR knockout methods. Related scientific discoveries and technical development in knockdown and knockout techniques can be considered when investigating the evolution of gene essentiality. For example, Green *et al* reported unexpected dsRNA construct landing site (40D3) of a public RNAi library and its phenotypic consequence [26]; Kondo *et al* investigated phenotypic consequences of newly added RNAi triggers into RNAi libraries [27], although its knockout analyses of new essential genes were re-examined by a recent thorough phenotypic analysis, which revealed essential functions in early development in tested young genes [17].

We will present in this report our recent experiments and computational analyses, examining a few important issues raised in recent years that we find to be generally relevant for the detection of the phenotypic effects of genes, particularly of those that recently originated. We conducted following analyses of newly raised technical problems and scientific issues: 1) the repeatability of knockdown analysis for testing essentiality phenotypes; 2) an evaluation of the knockdown efficiency distribution in RNAi experiments; 3) an understanding of the differences between different RNAi libraries in phenotyping large samples of new genes for viability effects; 4) a detection of compensation effect which may compromise detection of effects of CRISPR mutants in new gene duplicates; 5) a detection of developmental essential effects in a larger sample of new genes that are shown to be also in a higher resolution for their *Drosophila* lineage distribution based on our recent dating gene ages [15] than that previous analysis [25]. Our data, with additional evidence published recently by our group and others, provide ample and strong evidence to further support a concept suggested by the fitness effect analysis of new genes in *Drosophila*: new genes have quickly evolved essential functions in viability during development. Meanwhile, both the technique and the data we created regarding the RNAi knockdown analyses of repeatability, efficiency, and different libraries and detected compensation effect for highly similar gene duplicates will offer wide and valuable reference for detection of phenotypic effects of genes in general.

## Results

### High reproducibility of RNAi knockdown for detecting lethal phenotypes

**Fig 1:**
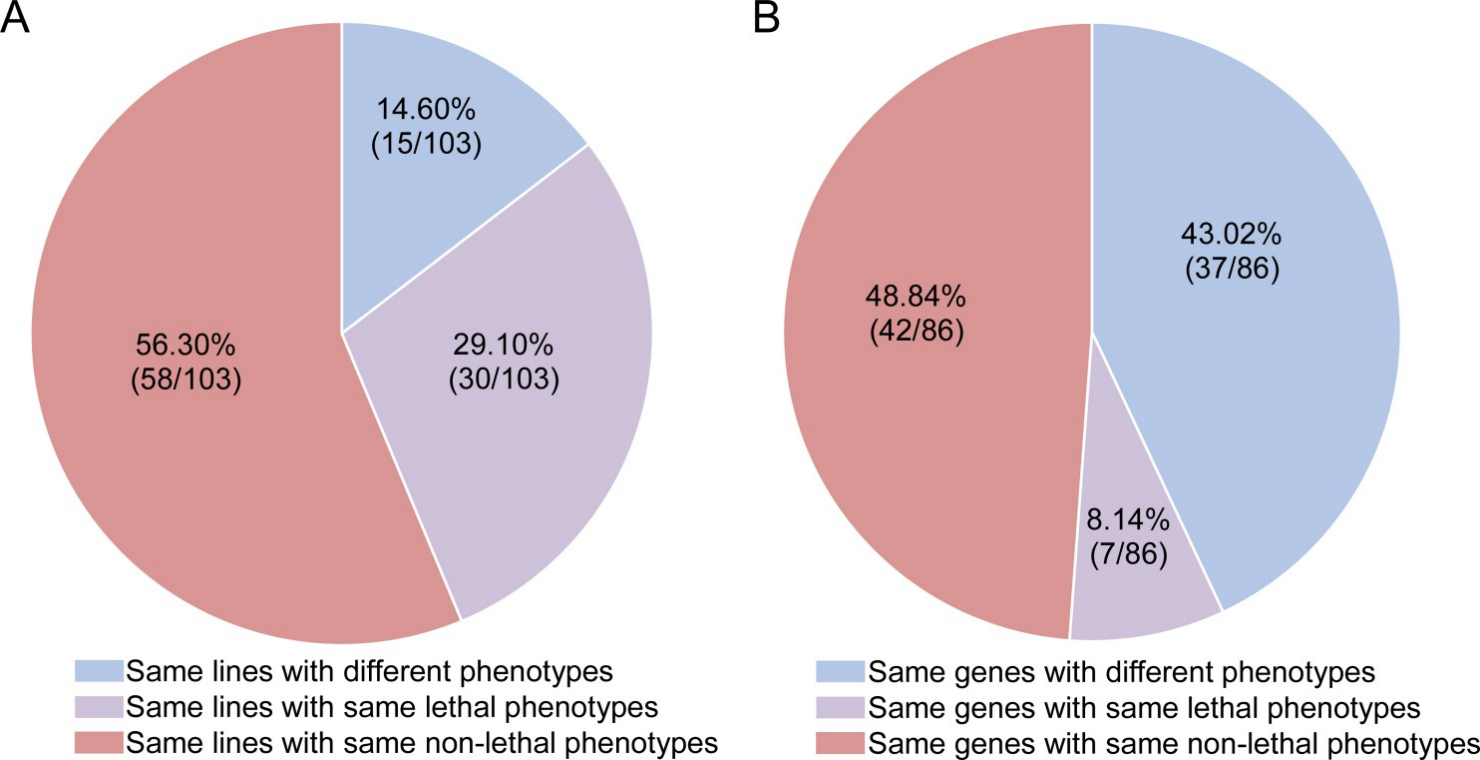
The reproducibility analysis of RNAi experiments by comparing two groups of independent experiments by [25] and [28]. A. Phenotypes of same 103 RNAi lines analyzed by [25] and [28] using same lines; B. Phenotypes of 86 same new genes knocked down by two different drivers or the same drivers with different insertion sites. The old drivers detected 29 genes as lethal while 57 non-lethal; the new drivers detected 20 genes as lethal while 66 non-lethal.

We investigated the consistency of RNAi experiments with the same lines and the same drivers in different laboratories, conditions, and years. Zeng *et al* independently screened 16,562 transgenic RNAi lines using an Act5C-Gal4 driver to detect the lethality of 12,705 protein-coding genes (~90% of all annotated coding genes) in their study of intestinal stem cell development and maintenance [28]. Their dataset included RNAi lines targeting the same 103 genes that were measured for lethality by Chen *et al* [25]. Chen *et al* and Zeng *et al* obtained the same phenotypes for 88 (85.4%) genes, including 30 (29.1%) of the lethal phenotype and 58 (56.3%) of non-lethal phenotype (Fig 1A, S1 Table). These data suggest that despite differences in independent observers, lab environments, and years to conduct experiments, the vast majority of RNAi knockdown experiments are reproducible for phenotyping lethality and non-lethality.

We also tested consistency between RNAi lines with different RNAi drivers (called new drivers) or same drivers in different genome positions. Specifically, the datasets of Chen *et al* and Zeng *et al* shared 86 new genes in knockdown experiments, mostly (81.4%, 70) with different RNAi drivers and fewer (18.6%, 16) same drivers in different genome positions (S2 Table). This dataset showed that: 7 genes were consistently lethal; 42 genes were consistently non-lethal; and 37 genes have different phenotypes (Fig 1B). Thus, the two groups with different drivers or same drivers with different positions reveal that a majority of new genes (57.0%, 49) show the same phenotypes.

### Low knockdown efficiency and better GD library than KK library

We considered an additional factor in RNAi knockdown, sensitivity, in the two widely used RNAi libraries: the Vienna Drosophila Resource Center’s (VDRC’s) GD and KK libraries [29]. The GD libraries were constructed using P-elements to randomly insert hairpin RNAs (average 321bp) into the genome targeting individual genes, while the KK library inserted constructs carrying hairpin RNAs (average 357bp) into a specific landing site by ΦC31-mediated homologous recombination. All KK lines carry an insertion at 30B3, but a proportion (23–25%) also carry an insertion at 40D3 (*tio* locus) that results in pupal lethality when using constitutive drivers like Act5C-GAL4 [26,30]. Unless specified, no lines discussed below contain 40D3 insertions.

**Fig 2:**
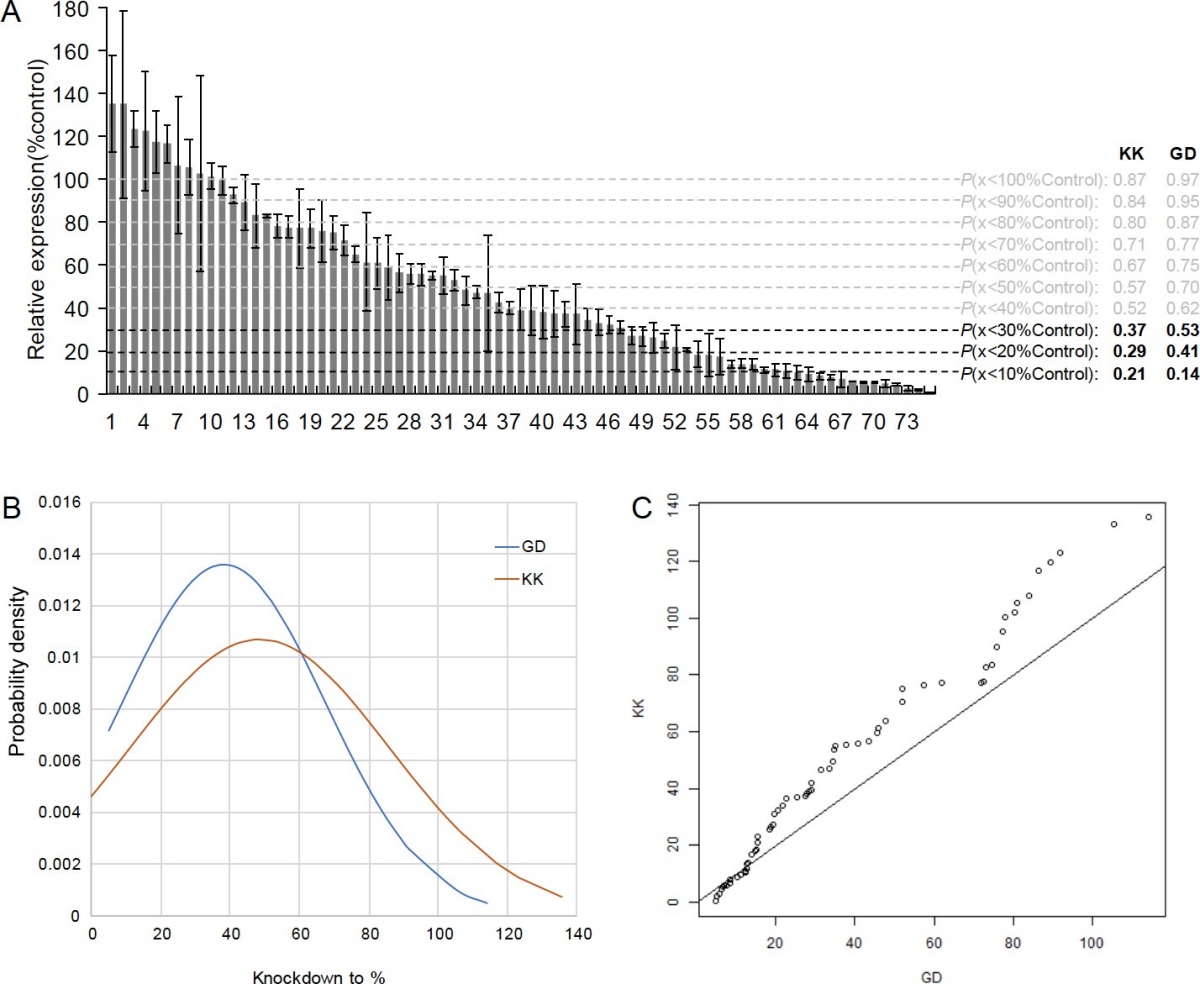
Knockdown efficiency in the KK and GD libraries revealed GD lines have significantly higher knockdown efficiency than the KK lines. A. The knockdown efficiency of the 75 KK lines was measured, compared to the expression of the wild-type control and the standard deviation is calculated from the measurement of three repeats; P refers to proportion of genes with the expression lower than a certain threshold while the values of KK lines are generated in this work and that of GD lines are extracted from [29]. B. The Z-score distributions of knockdown efficiency of KK and GD lines. z-score means the number of standard deviations from the mean. z = (x–μ)/σ, x is the value to be standardized, μ is the mean, σ is the standard deviation. 38.1% and 48.6% are average knockdown-to-percent of GD and KK lines, respectively. C. The Q-Q Plot (quantile-quantile plot) between KK and GD lines. Q-Q plot is a probability plot which is used here for comparing probability distributions between knockdown-to-percent of KK and GD lines by plotting their quantiles against each other. Those Q-Q plots don’t fit the best fitting straight line, which indicate that the knockdown efficiency distribution between KK and GD lines are different.

Given the intrinsic different designs of GD and KK libraries, we hypothesized that they have different false negative or false positive rates, which cause the inconsistency shown in Fig 1B. Only GD lines were examined previously, and they have a high false negative rate (39.9%) but low false positive rate (<2%) [29]. The high false negative rate is likely caused by insufficient target gene knockdown, while false positives may be due to off target effects [29]. We thus tested the knockdown efficiency of 75 KK lines targeting randomly selected 75 young genes (S3 Table, Fig 2A). We found that the knockdown efficiency of KK lines is generally lower than the efficiency of 64 GD lines as previously reported [29]. Specifically, using the same driver (Act5C), we found that in general, GD lines have significantly higher knockdown efficiency than KK lines, as shown by the knockdown expression as the percentage of the control expression (Fig 2A). That is, the KK lines have an average knockdown efficiency as 48.6% of control expression while the GD lines show an average efficiency as 38.1% (Fig 2B and 2C, *t*-test *P* = 0.031). Notably, the expression reduction to 50~60% level of the wide-type level was observed to have no significant fitness loss due to widespread haplosufficiency [31,32]. Detecting any fitness effect may be expected when the expression drops to a lower level, for example, 20~30% or lower of the control expression. In this range of knockdown efficiency, we observed that only 29% of KK lines but 41% of GD lines reduced target expression levels to ≤20% of control levels; 37% of KK lines but 53% of GD lines were seen to reduce target expression levels to ≤ 30% of control levels (Fig 2A). Thus, based on knockdown efficiency assay of 75 KK RNAi lines derived by Act5C, these observations derive two expectations: firstly, most of RNAi knockdown have so low efficiency that no phenotypic effects can be expected; secondly, GD lines, having a higher knockdown efficiency than KK lines, should have a higher power in detecting lethal phenotypes as shown in the next section.

**Fig 3:**
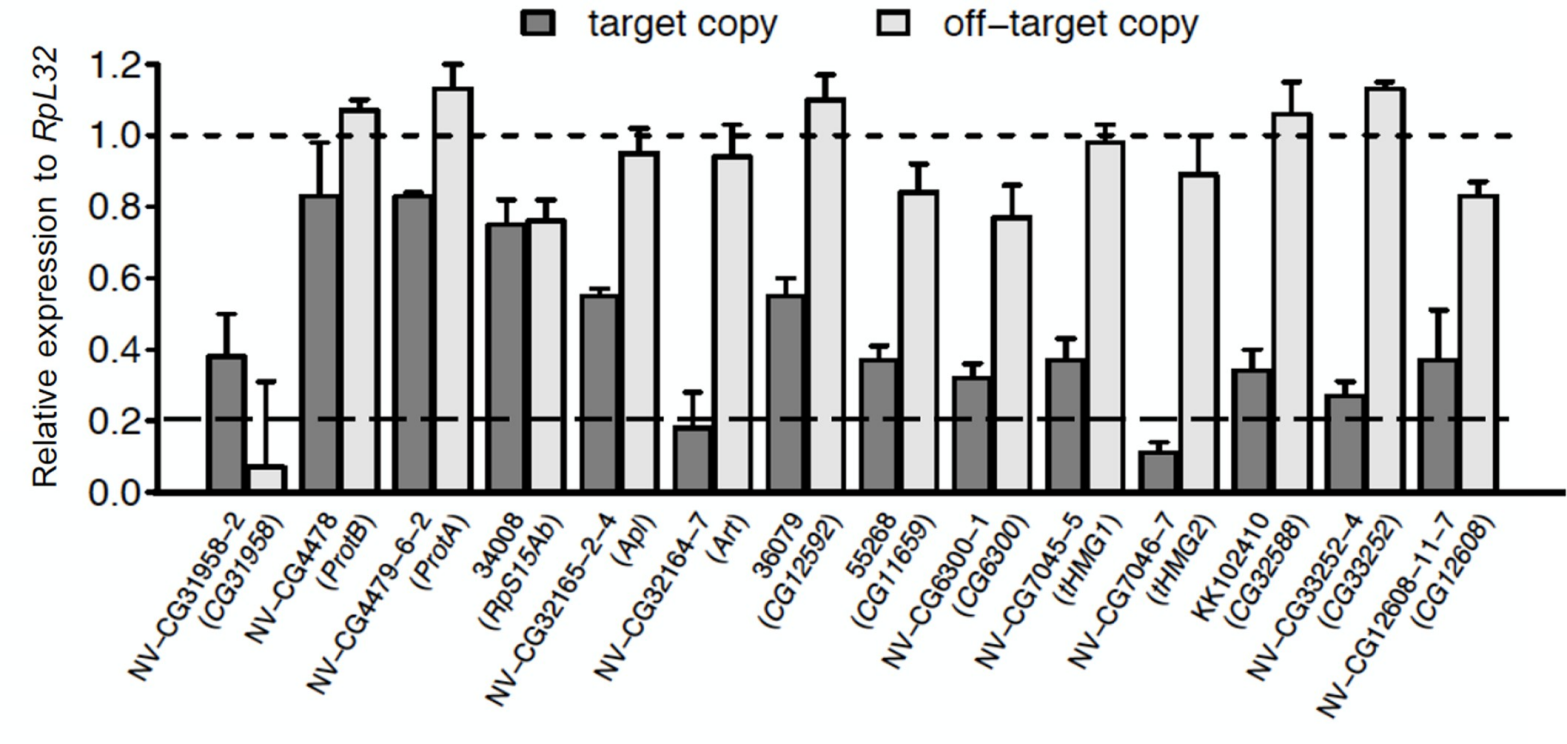
Experimental comparison of the efficiency and off-target effects explain the conservative nature of RNAi knockdown experiments and limited off-targets propensity. For each young duplicate gene pair specific for *D*. *melanogaster* and *melanogaster* species complex, we examined their expression intensity relative to the wide type control (relative expression level to WT normalized by *RpL32*) in whole body flies with qPCR. Only two lines likely produce off-target effects (NV-CG31958, 34008 (the TRiP line)), for both of which the expression of paralog is down-regulated to similar or even lower level compared to the corresponding gene of interest. However, the 34008 line have so low knockdown efficiency that no phenotypic effects can be expected. The standard deviation is calculated based on three replicates.

To estimate false positive rate of KK lines, we constructed 10 randomly chosen new KK lines targeting one member of a young duplicate gene pair, in addition to one KK line and 3 TRiP lines (Transgenic RNAi Project, BDSC, Materials and Methods). The rationale is that for each gene of interest its paralog is the most likely off target. The same rationale was also followed by [29] when false positive rates of GD lines were estimated. We measured the knockdown efficiency and estimated off-target effects using these 14 lines with qPCR experiments in adult whole bodies (Fig 3). We found that two lines likely produce off-target effects (*NV-CG31958*, *34008* (the TRiP line)), for both of which the expression of paralog is down-regulated to similar or even lower level compared to the corresponding gene of interest. Twelve other lines have significantly higher target effects than off-target effects, among which 10 genes reduced activity to 20–80% expression level of the control (7 genes reduced activity to 20–40%) and only two genes (*CG32164*, *CG7046*) reach≤20% of control levels. Thus, if we take 20% as the cutoff of efficient knockdown, only *CG31958* could be counted as the false positive, and *CG32164* and *CG7046* be counted as the true positives. Collectively speaking, the off-target effects are rare while insufficient knockdowns are pervasive.

These experiments detected an interesting variation of knockdown efficiency among different drivers where newer KK lines have lower efficiency and thus higher false negatives compared to older GD lines. Thus, if a newly constructed RNAi driver is added to the phenotypic analysis, insufficient knockdown is also introduced with a high probability, suggesting the new RNAi driver with the low knockdown efficiency is not revealing for a gene’s essentiality.

### Both GD and KK libraries detected similar proportions of lethality between new and old genes and a higher proportion of lethality in GD

We first investigated differences in measured lethality between the KK and GD libraries used in Chen *et al* [25]. To control for the confounding effect of *tio* insertion in the KK lines, we genotyped these lines using PCR-amplification and found that out of 153 KK lines we collected (S4 Table), 47 (30.7%) had two landing sites and 6 (3.9%) had only 40D3 landing site (the confounding site) [26], which all showed lethal phenotypes. Using the recombination approach [26], we recovered 41 of the 47 lines into the lines that have only the 30B3 site. The RNAi knockdown of 140 KK lines carrying insertions only at 30B3 identified 12 genes (8.6%) with lethal phenotypes. Meanwhile, 12 new genes in GD lines for 59 new genes (20.3%) were detected to have lethal knockdown effects [25], significantly higher than the KK libraries (*P* = 0.0112, Fisher’s Exact Test). As aforementioned, this difference is likely due to higher false negative rate of KK lines (Fig 2).

**Fig 4:**
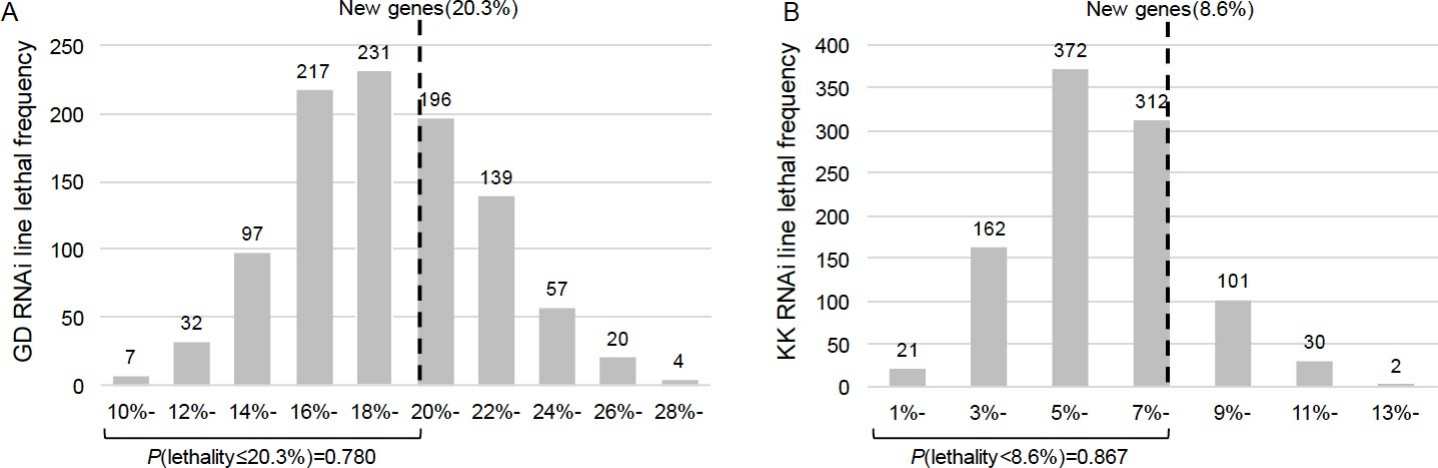
Comparison of proportions of lethality between new genes and old genes in GD lines (A) and KK lines (B) suggests that in both GD and KK lines, new genes have an equally high probability to be lethal as old genes. By using the essentiality data of 10,652 old genes provided by VDRC (https://stockcenter.vdrc.at/control/library_rnai) that were in branch 0 [15], we characterized the statistical distribution of essential old genes. Since old genes are much more abundant than new genes, we independently sampled 1000 times of old genes with the same number (150) of new genes and then plotted the distribution of proportion of essential genes as histograms. In the GD library, the probability to obtain a proportion of essential new genes equal or lower than 20.3% is 0.780. Meanwhile, in the KK library, the probability to observe a proportion of essential new genes equal or lower than 8.6% is 0.867. These analyses of GD and KK libraries reveal similarly that the proportions of new and old genes with lethal phenotypes are not statistically different.

By using the essentiality data of 10,652 old genes provided by VDRC (https://stockcenter.vdrc.at/control/library_rnai) that were in branch 0 [15], we characterized the statistical distribution of essential old genes (Fig 4). We independently sampled 1000 times, with each randomly sampling 150 old genes and calculating the proportion of essential ones. We found that in the GD library, the probability to obtain a proportion of essential new genes equal or lower than 20.3% is 0.780. Meanwhile, in the KK library, the probability to observe a proportion of essential new genes equal or lower than 8.6% is 0.867. These analyses of GD and KK libraries reveal similarly that the proportions of new and old genes with lethal phenotypes are not statistically different. We note here that due to the low knockdown efficiency in GD library, despite higher than KK library, the actual proportion of essential genes can be higher than 20.3% as measured by GD library.

### Genome-wide gene-dating in *Drosophila* phylogeny reveal that new genes evolve essential functions in development quickly

Further analysis of gene essentiality data in a recent version of VDRC libraries detected with increased resolution the proportions of essential genes in six detectable ancestral stages of *D*. *melanogaster*. We reported the analysis of the GD library, which has a significantly higher knockdown efficiency than the KK library. From several new gene duplicate datasets of *Drosophila* [33–35], we chose two recent datasets to compare [15,27](S1 Fig, also see the comparison in S1 File) because they are more updated and also used additional gene-synteny information from multiple species genomes besides considering substitution rates among paralogous and orthologous copies. Due to the better quality (S1 Fig) of GenTree and more complete types of new genes including DNA-based and RNA-based duplicates and orphan genes, we used its gene dating results for examining the evolution of essentiality in all *Drosophila* genes. In total, 11,354 genes (72% of 15,682 genes in the species, Ensembl 73) have been identified phenotype for their lethality or non-lethality, including 702 *Drosophila* genus specific genes (66% of 1,070 detected *Drosophila*-specific genes) [15,20,34,36] and 10,652 genes that predated the *Drosophila* divergence 40 Mya.

**Fig 5:**
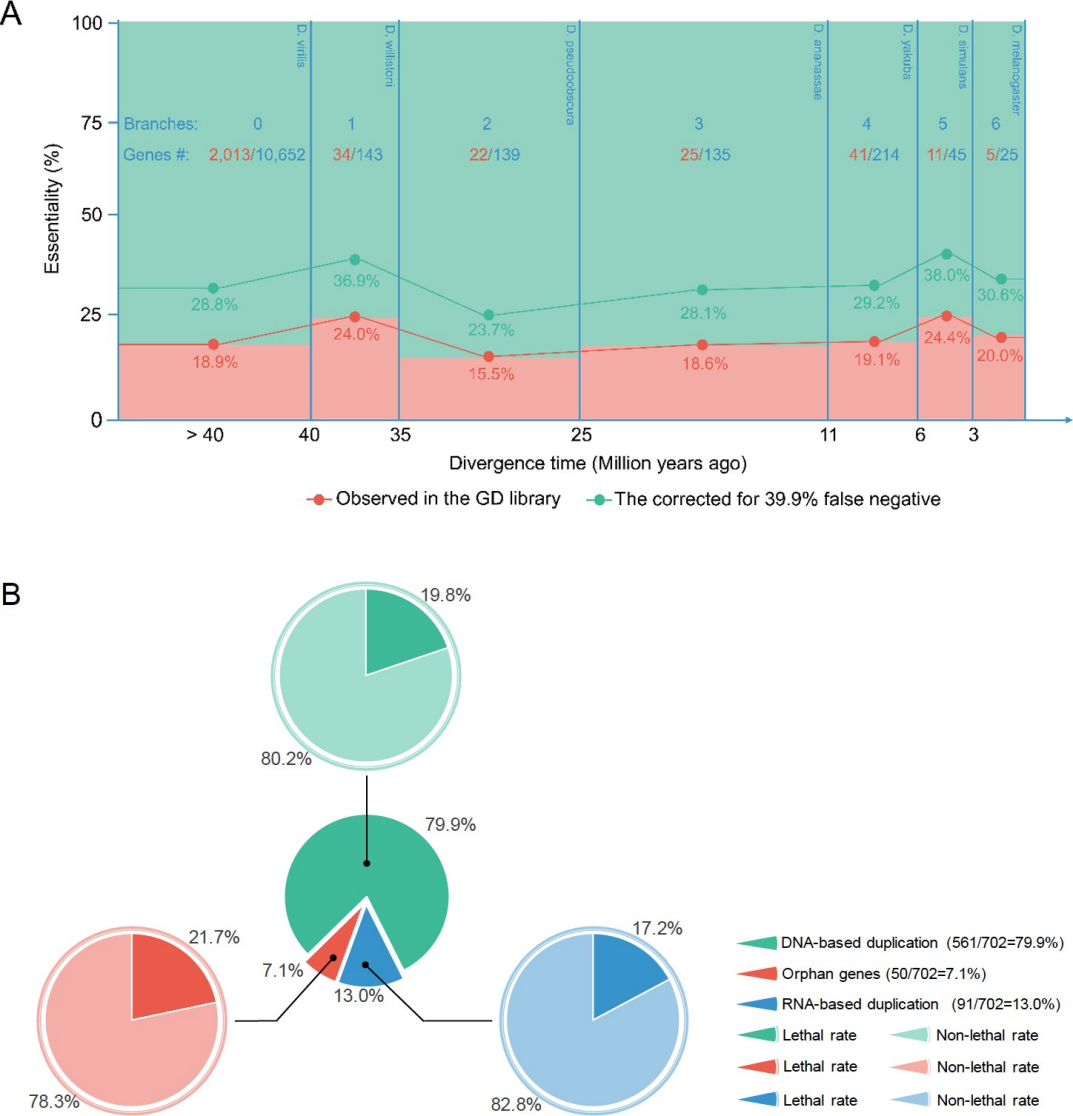
Lethality proportion of 702 *Drosophila*-specific genes. A. Lethality proportion of 702 *Drosophila*-specific genes in 6 ancestral stages of extant *D*. *melanogaster*, compared to the lethality proportion of 10,652 genes older than 40 Mya. No stages show an essentiality proportion significantly different from that of old genes (0.189). B. Lethality proportion of 702 Drosophila-specific genes based on three origin mechanism catalogs. No catalog shows a lethality proportion significantly different from that of old genes (0.189).

We parsimoniously mapped the 702 *Drosophila*-specific genes on the six ancestral stages by examining their species distribution in the *Drosophila* phylogeny [15] (Fig 5A). Of the 702 genes, 19.7% (138) are directly observed to be essential, similar to the proportion of essential old genes, 18.9% (*P* = 0.6212, Fisher’s Exact Test). We considered a low knockdown efficiency as shown by the 47% of GD lines whose knockdowns are expressed at the level of 30% or higher of the control (Fig 2A), suggesting that 47% of RNA lines are invalid for the testing and should be subtracted from the total tested lines.

Thus, the actual proportion of essential genes can be estimated by correcting for the bias of false positives (Fp) and false negatives (Fn) by following formula:

$$Corrected\;proportion\;of\;essential\;genes=\left[E–\left(T\cdot F_{p}\right)\right]/\left[T–\left(T\cdot F_{n}\right)\right]$$

Where E and T are observed number of essential genes and total number of genes examined, respectively. F_p_ was measured as 1.6% [29] while F_n_ as 47% as estimated above or 39.9% as measured previously [29]. Thus, the estimated proportion of essential genes after correcting false positives and false negatives can be as high as 36.5% for the estimated false negative rate of 47% in this study. The corrected proportion can be also as high as 32.2% given the previously measured false negative rate of 39.9%. Furthermore, all six stages show a stable proportion of essential genes; none of the proportions is statistically different from the proportion of old genes (Fig 5A). Meanwhile, lethal rates of new genes which belong to three origin mechanism categories (DNA-based duplication, RNA-based duplication and orphan genes) [15] also show no significant difference (Fig 5B). Interestingly, 21.7% of orphan genes, some of which might be *de novo* genes [20], are essential. These data add new insight into the evolution of essentiality in all ancestral stages: soon after genes originated and fixed in *D*. *melanogaster*, a stable and high proportion of new genes is essential throughout entire evolutionary process from ancient ancestors to the speciation of *D*. *melanogaster*.

### New gene duplicates show compensation effects in CRISPR frameshift mutants

It is now well documented that vertebrate cells such as mammalian cells or zebrafish cells recognize such aberrant mRNAs and compensate for their loss by increasing expression of genes with high sequence similarity, such as paralogs in zebrafish, worm and other organisms [37–41]. This has the effect of producing false negatives especially for recent duplicates that usually share high sequence similarity with parental duplicate copies. We detected a similar compensation effect in new gene duplicates in *Drosophila*.

**Fig 6:**
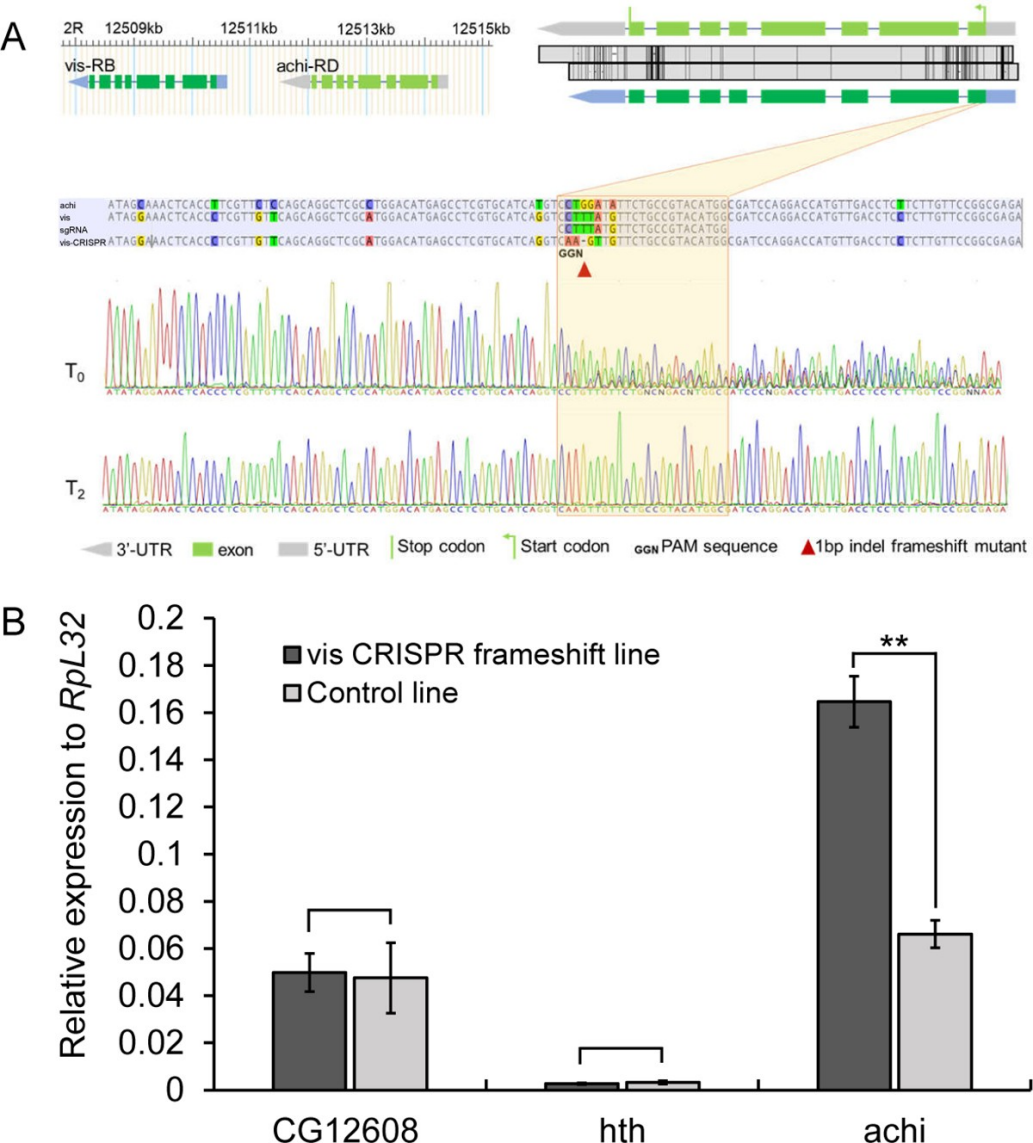
CRISPR/Cas9 frameshift mutant could induce compensatory effect in *Drosophila*. A. Design of CRISPR/Cas9 mutant. We targeted a randomly chosen young gene, *vis*, which emerged via duplication of *achi* in the common ancestor of *melanogaster* species complex. The genomic arrangement of two genes are shown in the upper left panel with the boxes referring to exons and connecting lines as introns. The pair shares a high sequence identity (0.92) in their 9 exons, which is schematically shown in the upper right panel. The middle panel shows the diverged site between *vis* and *achi*, which was chosen to design a short guide RNA (sgRNA) specifically targeting *vis*. The mutation (CTTTA→AAGT) was marked with a red triangle. The raw sanger sequencing data for the initial generation (T0) and the second generation of offspring (T2) was shown. B. The compensation effect of *achi*. In the frameshift mutant of *vis*, *achi*’s expression is significantly increased (P = 0.0003). By contrast, the unrelated *CG12608* and the remotely related *hth* did not show any significant upregulation. *RpL32* was used as a control as in [32].

We induced a one-nucleotide deletion using CRISPR/Cas9 into the ORF region of *vismay* (*vis*), a *D*. *melanogaster*-specific gene duplicated from a parental gene, *achintya* (*achi*), 0.8 Mya, with a nucleotide similarity of 92% between the two copies. We found that *achi* in the *vis* mutant was significantly upregulated whereas a randomly selected unrelated gene *CG12608* and the distantly related gene *hth* (nucleotide similarity of 45%) to *vis*, is not impacted by the *vis* mutation (Fig 6). Although the generality of the association between the *vis* mutation and enhanced expression level of its highly similar duplicate copy has yet to be further tested, its implication to test phenotypic effects of new gene duplicates is clear: the CRISPR knockouts do not offer gold-standard for detecting phenotypic effects.

## Discussion

Our experimental and data analyses yielded fresh insights into the conceptual and technical issues raised with the progresses in understanding of evolution of gene essentiality. We showed that the repeatability of *Drosophila* knockdown experiments from RNA libraries between independent researchers separated by several years is as high as 85.4%, a level inspirable for using the technique to detect phenotypic effects of genes.

On the other hand, we also found that the knockdown efficiency is generally low with publicly available RNAi libraries leading to a high false negative rate. This reveals a high conservative property of the technology, suggesting that actual proportion of essential genes is higher than the measured proportion. A failure to understand this property of RNAi knockdown erroneously led to a confusion of false negative as false positive in detection of essential phenotypes [27] (S1 File). Our genome-wide analysis of essentiality in the *Drosophila* phylogenetic tree reveals that the proportion of essentiality with the proportions of new genes are not significantly variable with evolutionary time periods as short as 3 million years to 14 million years. These genes generated within the *sophophora* subgenus lineages (<35 million years) are as similarly frequent as the older genes dated for more distantly diverged *Drosophila* lineages or the ancient genes in non-*Drosophila* ancestors.

Detailed case analyses for new gene functions provide several lines of evidence in support of the essentiality of several new genes in development. First, Ross *et al* reported a stepwise neofunctionalization evolution in which a centromere-targeting gene in *Drosophila*, *Umbrea*, was generated less than 15 Mya [19]. Both RNAi knockdown, rescue experiments and P-element mediated gene knockout revealed that *Umbrea* evolved a species-specific essentiality to target centromere in chromosome segregation [19,25]. Second, Lee *et al* recently detected stage-specific (embryos/larvae/pupa) lethality associated with RNAi knockdown and CRISPR knockout in *Cocoon*, a gene emerged 4 Mya in the common ancestor of the clade of *D*. *melanogaster-simulans* [18]. These data show that *Cocoon* is essential for the survival at multiple developmental stages, including the critical embryonic stage. Third, P-element insertion/excision experiments show the essentiality of *K81* as a paternal element in early development. This gene only exists in the *Drosophila melanogaster*-subgroup species that diverged 6 Mya [42]. Fourth, Zeus, a gene that duplicated from the highly conserved transcription factor *CAF40* 4 Mya in the common ancestor of *D*. *melanogaster* and *D*. *simulans* rapidly evolved new essential functions in male reproductive functions, as detected in the null mutants and knockdown [43,44]. Fifth, A pair of extremely young duplicates, *Apollo* (*Apl*) and *Artemis* (*Arts*), was found to have been fixed 200,000 years ago in *D*. *melanogaster* populations [32]. CRISPR-created gene deletions of these genes showed that both evolved distinct essential functions in gametogenesis and *Apl* critical function in development.

Finally, in a comprehensive functional and evolutionary analysis of the ZAD-ZNF gene family in *Drosophila* [17], 86 paralogous copies were identified with phenotypic effects detected by knockdown and knockout in *D*. *melanogaster*. It was found that the proportion (17/58 = 29.3%) of lethal copies in old duplicates (>40 Mya) and the proportion (11/28 = 39.3%) of lethal copies in *Drosophila*-specific duplicates (<40 Mya) are statistically similar. Development analysis of two recently duplicated copies, *Nicknack* and *Oddjob*, using thorough knockdown and knockout analyses provided compelling evidence that these recent duplicates are essential in development, falsifying the new gene knockout results of Kondo *et al* [27].

A well-supported hallmark of young new genes is their male-biased expression pattern (often testis/accessory gland specific) [45–47]. However, new genes in *Drosophila* quickly evolved essential functions in viability with an approximately equal lethal rate with old genes (Fig 6A). We further compared the lethal rates between the two sex-biased subgroups, especially male-biased genes, based on the same phenotypic data with Fig 6A and found that the lethal rate of those male-bias genes is significantly higher than non-male-bias genes. This is consistent with our previous observation in a pair of extremely young genes, *Apollo* (male-biased expressed) and *Artemis* (female-biased expressed), that were created just 200,000 years ago in *D*. *melanogaster* [32]. Both *Apollo* and *Artemis* showed lethality in development whereas the *Apollo* showed even stronger effects. These observations reveal a new and interesting phenomenology that may deserve attention: the development is coupling with the sex-specific expression in the importance of gene functionality.

Overall, these data from present and previous studies challenge a conventional belief in the antiquity of important gene functions in general [21,24,48,49] and in development in specific [22–23].

## Materials and methods

### RNAi strain construction

Since species-specific new genes are under-represented in public RNAi lines, we generated new RNAi lines following [29]. Briefly speaking, we designed RNAi reagents using the E-RNAi server (http://www.dkfz.de/signaling/e-rnai3/) and kept constructs with all possible 19-mers uniquely matching the intended target gene and excluded designs with >1 CAN repeat (simple tandem repeats of the trinucleotide with N indicates any base) [50]. Constructs were cloned into pKC26 following the Vienna Drosophila Resource Center’s (VDRC’s) KK library strategy (http://stockcenter.vdrc.at, last accessed 2 February 2016). We introgressed the X chromosome from Bloomington Drosophila Stock Center line 34772, which expresses ΦC31 integrase in ovary under control of the *nanos* promoter, into the VDRC 60100 strain. Strain 60100 carries attP sites at 2L:22,019,296 and 2L:9,437,482 [26]. We ensured that our RNAi constructs were inserted only at the 2L:9,437,482 site using PCR following Green *et al* [26]. RNAi constructs were injected into the 60100-ΦC31 at 250 ng/μL. Surviving adult flies were crossed to sna^Sco^/CyO balancer flies (BDSC 9325) and individual insertion strains were isolated by backcrossing.

### RNAi screen

We knocked down target gene expression using driver lines constitutively and ubiquitously expressing GAL4 under the control of either the *Actin5C* or *αTubulin84B* promoter. We replaced driver line’s balancer chromosomes with GFP-marked chromosomes to track non-RNAi progeny. Control crosses used flies from the background strains 60100-ΦC31, 25709, or 25710 crossed to driver strains. Five males and five virgin driver females were used in each cross. Crosses were grown at 25°C, 40% - 60% humidity, and a 12h:12h light: dark cycle. F1 progeny were counted at day 19 after crossing, after all pupae had emerged. We screened F1 RNAi flies for visible morphological defects in 1) wings: vein patterning and numbers, wing periphery; 2) notum: general bristle organization and number, structure and smoothness; 3) legs: number of segments. We monitored survival of RNAi F1s by counting GFP and non-GFP L1, L3 larvae and pupae. We tested RNAi F1 sterility by crossing individual RNAi F1 flies to 60100-ΦC31 and monitoring vials for L1 production. Ten replicates for each sex for each line were performed.

### RNAi knockdown specificity and sensitivity

We sought to address two known problems of RNAi technology using RT-qPCR. First, since off-target effects are often discussed in RNAi experiments [29] we need to test whether target gene expression are specifically knocked down, although our constructs are computationally predicted to be specific. Second, since the RNAi knockdown is often incomplete [29], we need to estimate how many genes are adequately knockdown in expression. We targeted a random dataset of 14 *D*. *melanogaster*-specific genes. We collected qPCR primers from FlyPrimerBank [51]. For those genes not found in FlyPrimerBank, we took Primer-BLAST to design primers by specifically targeting a ~100 bp region of the gene (S5 Table). We confirmed primer specificity with PCR and Sanger sequencing.

We randomly selected 75 KK RNAi lines (no *tio* site insertion) to analyze their knock down efficiency. We cross these 75 KK RNAi lines with same driver which was used in Dietzl *et al* [29] for GD RNAi line knock down efficiency test. We extracted RNA from sets of 8 adult males (2~4 day old) in triplicate from each RNAi cross using TRIzol (Catalog# 15596–026, Invitrogen, USA), treated ~2 μg RNA with RNase-free DNase I (Catalog# M0303S NEW ENGLAND Biolabs, USA), then used 1 μL treated RNA in cDNA synthesis with SuperScript III Reverse Transciptase (Invitrogen, USA) using oligo(dT)_20_ primers. cDNA was diluted 1:40 in water before using 1 μL as template in 10 μL qPCRs with Universal SYBR Green Supermix (Catalog# 1725121, Bio-Rad, USA) and 400 nM each primer. Reactions were run on a Bio-Rad C1000 Touch thermal cycler with CFX96 detection system (BioRad, CA). Cycling conditions were 95°C for 30 sec, then 45 cycles of 95°C for 5 sec, 60°C for 30 sec, and 72°C for 15 sec. We normalized gene expression levels using the *ΔΔC*_*T*_ method and *RpL32* as the control [29,52]. We tested the specificity and efficiency (90%< qPCR Efficiency<110%) of qPCR primers using an 8-log_2_ dilution series for each primer pair [32].

### Testing compensation effects of new gene duplicates

We generated the frameshift mutation line of *vis* using the CRISPR protocol previously developed [32] but with one single sgRNA for one gene as Kondo *et al* did [27]. The sgRNA-*vis* primer below was synthesized (the underlined sequence):

5’-GAAATTAATACGACTCACTATAGGATGTACGGCAGAACATAAGTTTAA

GAGCTATGCTGGAA-3’;

We used the following sequence-specific qRT-PCR primers to test the compensatory expression of *achi*, the duplicate of *vis*. Two control genes including *CG12608* and *hth* were examined too. Since *vis*’s expression is largely testis-specific, we extracted RNAs from testis of mated 4-day males and used qRT-PCR with 3 replicates to assess the expression, as developed previously [32].

248bp:

Achi-RT1F: 5’-AAAGTGACAGGTTTCTCTGTTTG-3’;

Achi-RT1R: 5’-CTGATCCTCCTCCACGATGAC-3’.

237bp:

CG12608-RT1F: 5’-CATAGTGGGCACCTACGAG-3’;

CG12608-RT1R: 5’-TGCGAGAGTATGATCTGCGAC-3’.

92bp:

hth-RT1F:5’-CCTAGTCATGTATCGCCGGTC-3’;

hth-RT1R:5’-AGCGGATGTTCATAAATCGCA-3’.

Internal control:

113bp:

RpL32-RT1F: 5’-AGCATACAGGCCCAAGATCG-3’;

RpL32-RT1R: 5’-TGTTGTCGATACCCTTGGGC-3’.

## Supporting information

S1 Fig Summary of new gene candidates in the K-dataset and G-dataset. A. phylogenetic distribution of gene origination identified by the K-pipeline and the G-pipeline as shown in the two datasets. B. Evaluation of the two datasets based on individual gene analyses. The two datasets share 471 candidates (red). The G-dataset consist of 101 authentic candidates (deep blue) undetected in the K-dataset, 19 false positives (light purple), 18 dubious cases (green) and 45 cases not applicable for dating (sky blue). By contrast, the K-dataset includes 49 bona fide new gene candidates, 318 false positives, 102 dubious cases and 242 difficult cases. For the 318 false positive genes, 275 actually have orthologs in outgroup species; 32 noncoding or pseudogene models are taken as protein coding genes; 6 redundant entries of same genes are treated as different genes; 5 polycistronic coding genes are misdated. For possible reasons of false positive new gene caused by incorrect ortholog analyses: 1. existing orthologs not identified in the K dataset because of a different cutoff value in identifying an outgroup ortholog; 2. orthologs may not have been annotated in outgroup species; 3. genes without orthologs in adjacent outgroup species but actually having orthologs in more distant outgroup species. Note, the K-dataset mentions 1,182 genes in the main text, however its associated supplemental table includes 1,176 genes with 6 genes listed more than once.
(TIF)

S2 Fig Age dating between this work and Kondo et al. This Figure, following S1 Fig in this paper, adds specific information on how we classified genes into six major categories or dozens of subcategories. For more details, please refer to S1 File.
(TIF)

S3 Fig A representative difficult-to-date locus in the K-dataset. A. The syntenic view of Ste locus between *D*. *melanogaster* and *D*. *simulans* shows fragmented continuity. Due to its multiplicative nature, Ste locus is difficult to assemble. In the UCSC Net track, the most assembles can only reach level 2 of one-way syntenic mapping, rather than a better reciprocal syntenic mapping as level 1. B. Some orthologous region in *D*. *simulans* (lifted from *D*. *melanogaster*) is not anchored to the chromosome (X) and they are arbitrarily assembled as chrU. C. In *D*. *sechellia*, two scaffolds are assembled with the major scaffold super_20 spanning 200 kb, in contrast to the assembly of 15 kb for the orthologous region of *D*. *melanogaster*.
(TIF)

S1 Table 103 knockdown experiments repeated by two independent works [25,28]. Note, works from [25] classified phenotypes as lethal, semi-lethal and viable. Since there are only few genes deemed as semi-lethal, we merged them into lethal gene groups to simplify.
(XLSX)

S2 Table For 86 new genes with different RNAi drivers, the consistency between different drivers in [25] and [28] is listed. (XLSX)

S3 Table The knockdown efficiency data of KK library and GD library. (XLSX)

S4 Table The genotyping results of 153 KK lines, the corrected lines by recombination and knockdown results. (XLSX)

S5 Table Primers for 75 KK lines knockdown efficiency tests. (XLSX)

S6 Table The list of 471 genes with the exact ages across the G-dataset and the K-dataset. (This table consists of three sub-tables to show genes with the exact ages across the G-dataset and the K-dataset, genes with slightly younger ages in the K-dataset and genes with slightly older ages in the K-dataset, respectively).
(XLSX)

S7 Table The list of false negatives and false positives in the K-dataset. Since the Pan-*Drosophilid* age group in the K-dataset corresponds to the age group 0, 1 or 2 in the G-dataset (S1A Fig), we simply replaced the Pan-*Drosophilid* age group as 0/1/2 in the table if applicable.
(XLSX)

S1 File Supplementary Materials and Method. (DOCX)
