# Supplementary figures and images for: Genomic analyses of new genes and their phenotypic effects reveal rapid evolution of essential functions in Drosophila development
Source: PLoS Genet. 2021 Jul 9;17(7):e1009654. doi: 10.1371/journal.pgen.1009654 (PMC8270118; doi:10.1371/journal.pgen.1009654)

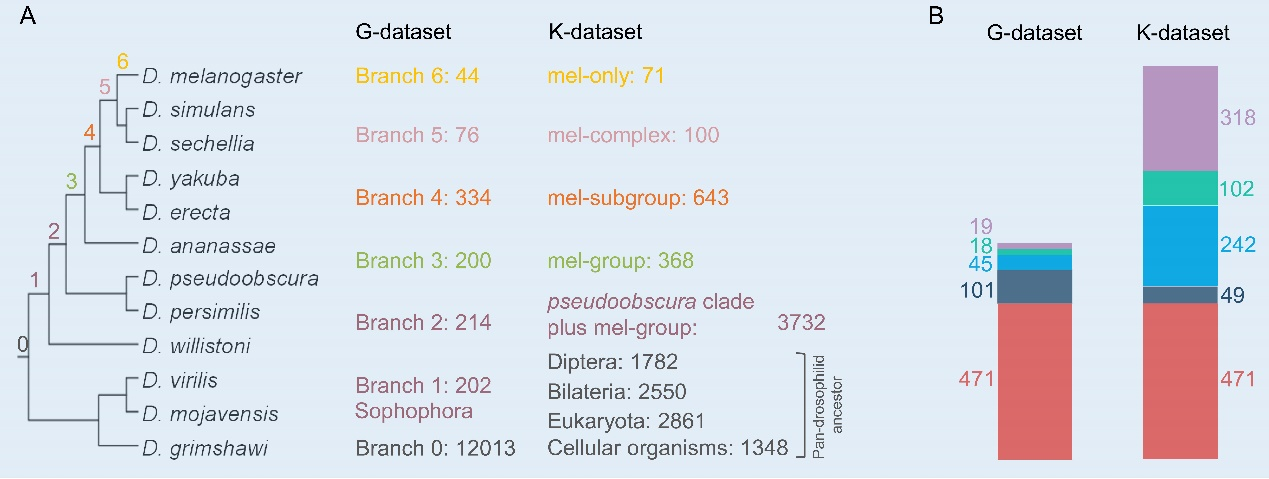

Supplement: S1 Fig — A. phylogenetic distribution of gene origination identified by the K-pipeline and the G-pipeline as shown in the two datasets. B. Evaluation of the two datasets based on individual gene analyses. The two datasets share 471 candidates (red). The G-dataset consist of 101 authentic candidates (deep blue) undetected in the K-dataset, 19 false positives (light purple), 18 dubious cases (green) and 45 cases not applicable for dating (sky blue). By contrast, the K-dataset includes 49 bona fide new gene candidates, 318 false positives, 102 dubious cases and 242 difficult cases. For the 318 false positive genes, 275 actually have orthologs in outgroup species; 32 noncoding or pseudogene models are taken as protein coding genes; 6 redundant entries of same genes are treated as different genes; 5 polycistronic coding genes are misdated. For possible reasons of false positive new gene caused by incorrect ortholog analyses: 1. existing orthologs not identified in the K dataset because of a different cutoff value in identifying an outgroup ortholog; 2. orthologs may not have been annotated in outgroup species; 3. genes without orthologs in adjacent outgroup species but actually having orthologs in more distant outgroup species. Note, the K-dataset mentions 1,182 genes in the main text, however its associated supplemental table includes 1,176 genes with 6 genes listed more than once. (TIF) [file pgen.1009654.s001.tif]

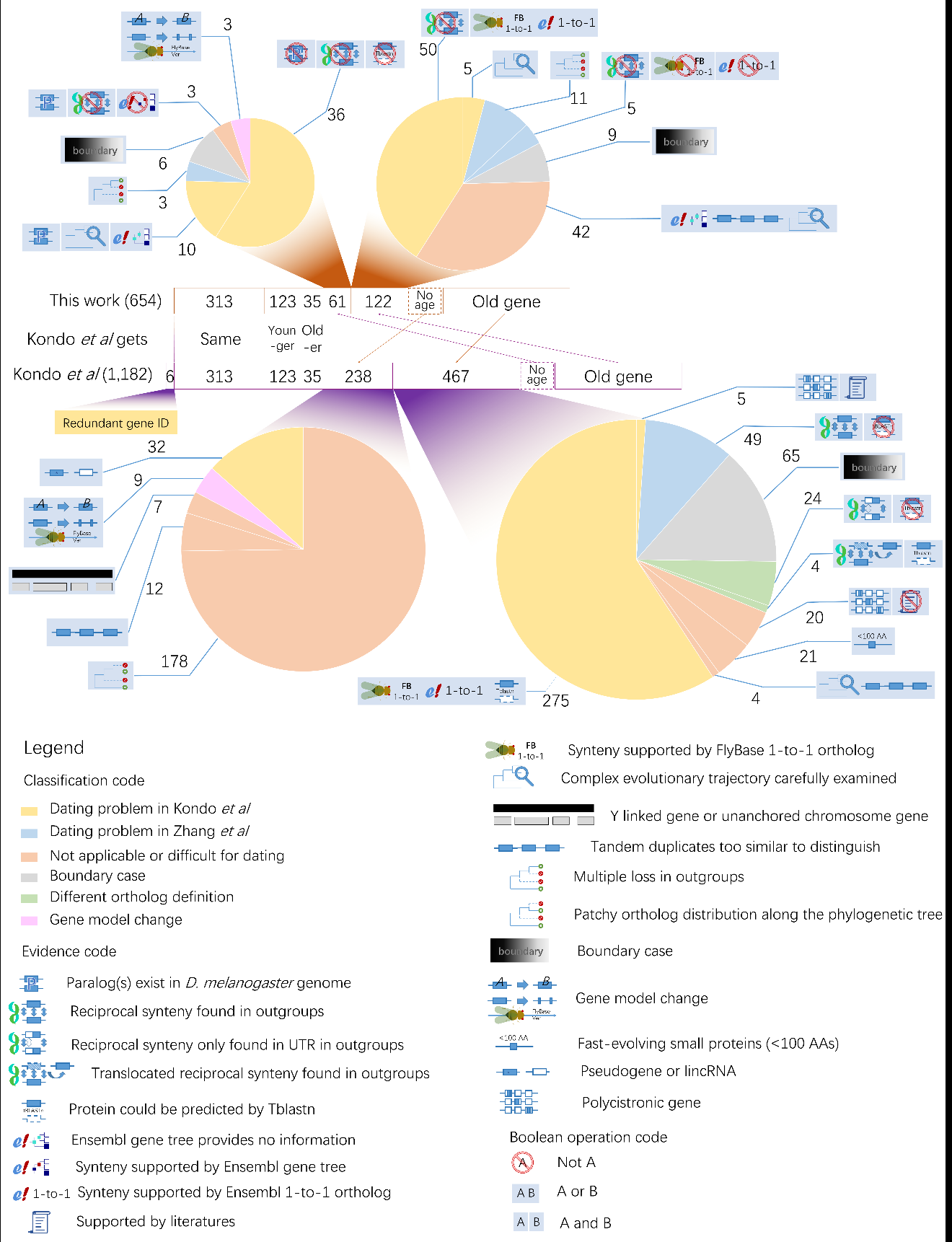

Supplement: S2 Fig — This Figure, following S1 Fig in this paper, adds specific information on how we classified genes into six major categories or dozens of subcategories. For more details, please refer to S1 File. (TIF) [file pgen.1009654.s002.tif]

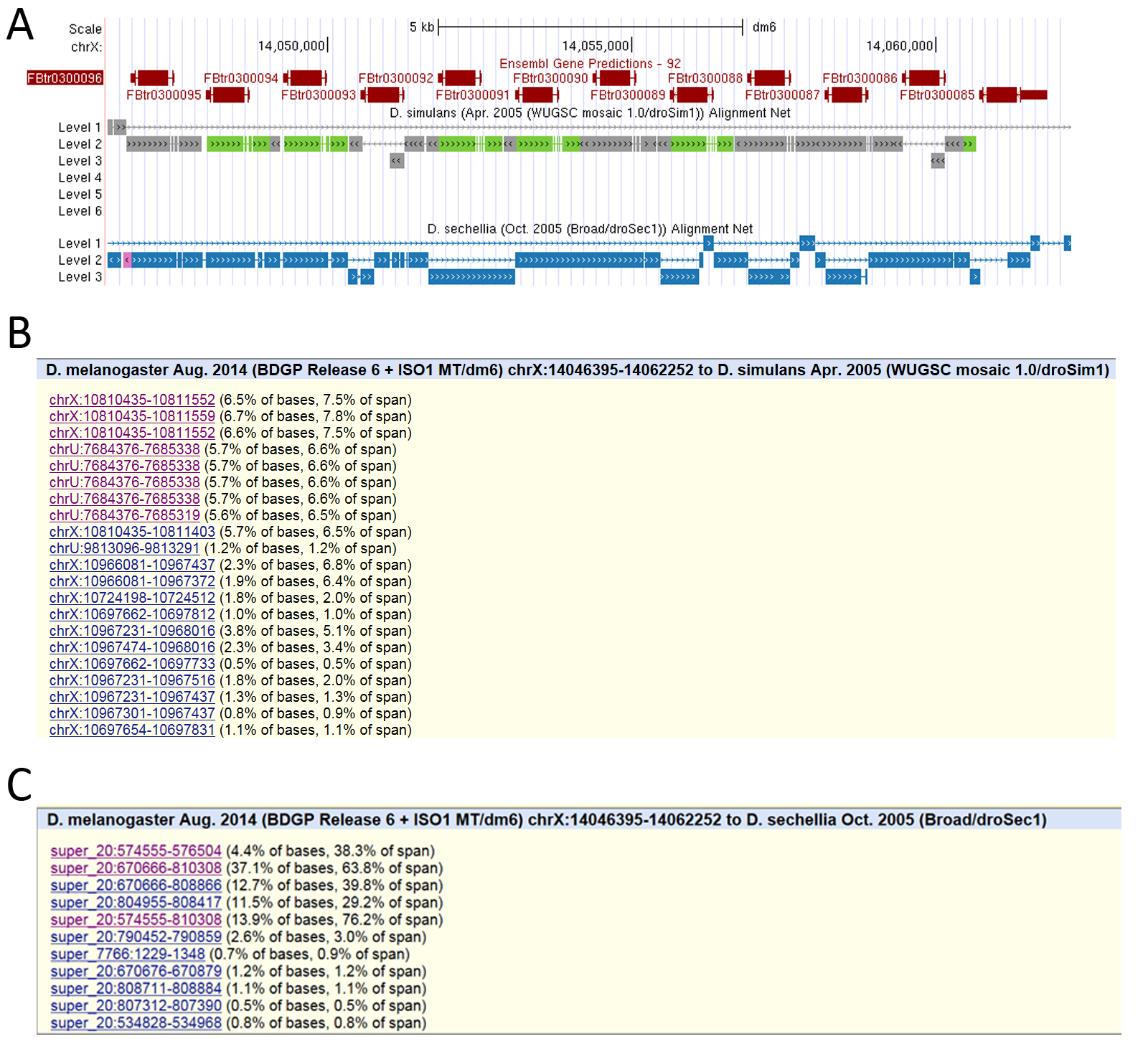

Supplement: S3 Fig — A. The syntenic view of Ste locus between D. melanogaster and D. simulans shows fragmented continuity. Due to its multiplicative nature, Ste locus is difficult to assemble. In the UCSC Net track, the most assembles can only reach level 2 of one-way syntenic mapping, rather than a better reciprocal syntenic mapping as level 1. B. Some orthologous region in D. simulans (lifted from D. melanogaster) is not anchored to the chromosome (X) and they are arbitrarily assembled as chrU. C. In D. sechellia, two scaffolds are assembled with the major scaffold super_20 spanning 200 kb, in contrast to the assembly of 15 kb for the orthologous region of D. melanogaster. (TIF) [file pgen.1009654.s003.tif]
