# Supplementary material for: Genomic analyses of new genes and their phenotypic effects reveal rapid evolution of essential functions in Drosophila development
Source: PLoS Genet. 2021 Jul 9;17(7):e1009654. doi: 10.1371/journal.pgen.1009654 (PMC8270118; doi:10.1371/journal.pgen.1009654)
Supplement: S1 File — (DOCX) [file pgen.1009654.s011.docx]

**Genomic Analyses of New Genes and Their Phenotypic Effects Reveal Rapid Evolution of Essential Functions in Drosophila Development**

Shengqian Xia1^§^, Nicholas W. VanKuren1^§^, Chunyan Chen2,3^§^, Li Zhang1, Clause

Kemkemer1, Yi Shao2,3, Hangxing Jia2,3, UnJin Lee1, Alexander S. Advani1, Andrea

Gschwend4, Maria D. Vibranovski5, Sidi Chen6, Yong E. Zhang2,3,7* and Manyuan Long1*

**1** Department of Ecology and Evolution, The University of Chicago, Chicago, Illinois, United States of America, **2** State Key Laboratory of Integrated Management of Pest Insects and Rodents & Key Laboratory of Zoological Systematics and Evolution, Institute of Zoology, Chinese Academy of Sciences, Beijing, China, **3** University of Chinese Academy of Sciences, Beijing, China, **4** Department of Horticulture & Crop Science, The Ohio State University, Columbus, Ohio, United States of America, **5** Department of Genetics and Evolutionary Biology, University of São Paulo, São Paulo, Brazil, **6** Department of Genetics, Yale School of Medicine, West Haven, Connecticut, United States of America, **7** Center for Excellence in Animal Evolution and Genetics, Chinese Academy of Sciences, Kunming, Yunnan, China

§Co-first authors

*Corresponding authors: mlong@uchicago.edu (MYL); zhangyong@ioz.ac.cn(YEZ)

**Supplementary Materials and Methods**

**Identification of *Drosophila*-specific genes by the age dating.**

Two new gene datasets (those genes with ages < 40 Million years ago, Mya) are available for *D. melanogaster*, which include the dataset of Kondo *et al* ([1], the K-dataset, the underlying pipeline as the K-pipeline) and the dataset we recently reported ([2]; [3]called as the GenTree Fly dataset, the G-dataset). In order to determine which dataset is more accurate and thus could be used in the downstream analyses, we estimated their qualities by performing systematic comparison. Kondo *et al* identified 1,182 new genes that postdated the split of *D. melanogaster* and *D. pseudoobscura* [1] (Branches 3~6, ~40 Mya(million years ago); S1A and S1B Fig). They inferred the ages of these genes by incorporating the UCSC DNA-level synteny information, homology information based on comparison of annotated proteins and RNA expression profiling. By contrast, we identified 654 new genes in this same evolutionary period (S1A and S1B Fig) using the same UCSC synteny information [4] and our maximum parsimony-based pipelines [2].

We investigated why the K-dataset was almost twice the G-dataset over the same evolutionary period. The K-dataset contained 471 new gene candidates in the G-dataset (471/654 = 72%) (S1B Fig, red), among which 313 are of the exact same ages while 158 show either younger (123 genes) or older (35 genes) (S6 Table). For the remaining 183 genes absent in the K-dataset, we found 101 as authentic new genes (S1B Fig, deep blue; S7 Table) after extensive validation by checking UCSC synteny information and four additional resources including the FlyBase ortholog annotation, Ensembl Metazoa homolog annotation, protein prediction in outgroup species and published literatures (see also Materials and Methods). This result indicates a high false negative rate in the K-dataset. By contrast, only 19 genes are old genes, which represent false positives in the G-dataset (S1B Fig, light purple). Then, for 45 genes (S1B Fig, sky blue), they are located in clusters of tandemly amplified genes or transposon-rich regions, where synteny is often ambiguous or difficult to build especially when outgroup species genomes are poorly assembled. Their ages are difficult to infer. Analogously, the final remaining 18 candidates (S1B Fig, green) are dubious, either with inconsistent topology between UCSC synteny information and Ensembl tree, marginal protein similarity between species or gene structure model changes.

We next examined the 711 (1182-471=711) new gene candidates unique to the K-dataset by examining phylogenetic distribution and their syntenic relationship with genes in various species. We could only confirm 49 authentic new genes, which represent false negatives of the G-dataset. By contrast, 318 out of 711 genes were incorrectly dated as new genes due to four problematic practices (S1B Fig; S7 Table lists the 318 false positives): 1) neglecting 275 that have orthologs in outgroup species; 2) taking 32 noncoding or pseudogene models as protein coding genes; 3) treating 6 redundant entries of same genes as different genes; and 4) misdating 5 polycistronic coding genes reported by the literatures. In addition, 242 (S1B Fig, sky blue) genes are located in repetitive regions. To be conservative, the G-dataset excluded these genes from dating. Then, the remaining 102 candidates (S1B Fig, green) are dubious in the age dating.

We conclude, based on above exhaustive evaluation, that the G-dataset is of much higher quality compared to the K-dataset: 1) the false negative rate and false positive rate of the G-dataset is estimated as 7.5% (49) and 2.9% (19), respectively; 2) The both parameters of the K-dataset are higher, 8.5% (101) and 26.9% (318), respectively; 3) the G-dataset only contains 9.6% (63) low-quality candidates (genes in repetitive or dubious categories), while the K-dataset consists of 29.1% (344) such candidates. Overall, 56% (662/1,182) new gene candidates in the K-dataset are either false positive or dubious.

***Comparison of the K-dataset and the G-dataset for Drosophila new genes.***

*Overall comparison scheme*

To our knowledge, although ages of duplicate gene/de novo gene have been estimated by many other independent groups [5-7], there is no genome-wide evaluation of all annotated gene ages in Drosophila. Specifically, around a decade ago, we took advantage of the syntenic genomic (DNA) alignment generated by the UCSC group and performed the genome-wide age dating for the first time in *Drosophila* [2]. At that moment, we compared our data to previous studies based on limited number of cases and discovered the general reproducibility across studies. The genome-wide dataset by Kondo *et al.* enabled a systematic large-scale comparison. We began with repeating our pipeline on FlyBase annotation v6.02 and identified 654 new genes originated on the branch toward *D. melanogaster* after the species split of *D. melanogaster* and *D. pseudoobscura* (S1 Fig). Concurrently, based on similar FlyBase release v6.13, Kondo *et al.* performed dating according to the same syntenic alignment of UCSC, which is further complemented by v6.02 protein-level BLAST search and filter with testis-specific expression [1]. With additional Dollo-parsimonious searches, they identified 1,182 new genes originated in the same period including *melanogaster*-group to *melanogaster*-only, which correspond to age group 3 to 6 in our analysis, respectively (S2 Fig). Since the FlyBase annotation version is similar (v6.02 vs. v6.13), only 12 entries out of the G-dataset and K-dataset are not comparable due to “Gene model change” (S2 Fig). They represent either expired models or new models in v6.13. Except them, all other genes can be compared across two datasets.

We found that 471 out of the 654 new gene candidates in the G-dataset are covered in the K-database by comparing the Ensembl IDs of these databases. Moreover, 313 (66%) genes show the exact same ages. Since manual curation needs extensive efforts, we did not examine why the remaining 158 genes show minor age difference. Instead, we subsequently only focused on those genes which show conflicting dating results, *i.e.*, included or excluded in new gene dataset across two studies. As a result, we classified the conflicting cases into six major categories, which can be further divided into around 20 more specific sub-categories (S2 Fig). We documented how we performed classification as below.

*Four independent information sources facilitate evaluations of two age datasets*

The challenges in the dating of gene age largely lie in the ambiguity of calling orthologs across outgroup species [8]. We found that the conflict of age dating was often due to the difference of DNA-level synteny and protein-level homology search. Specifically, for a gene of interest, *A*, the UCSC best-to-best synteny information shows that its ortholog is present in one outgroup species, *B*. However, the protein-level information may reveal an absence. The opposite scenario can occur too. In these conflicts, we turned to independent resources including FlyBase ortholog annotation, the homolog annotation and gene family tree provided by Ensembl Metazoa [9,10], protein prediction in outgroup species based on gene models of *D. melanogaster* and literatures. Specifically, FlyBase provided AAA (Assembly/Alignment/Annotation) syntenic ortholog annotation. If species *B* encodes a FlyBase annotated ortholog, gene *A* likely predated the species-split of *D. melanogaster* and *B*. Similarly, Ensembl Metazoa provided one-to-one best-to-best ortholog annotation. We used it like FlyBase. Finally, for some cases where synteny predicted orthologous regions of *B* do not harbor an annotated gene, we conceptually translated this region with the protein of *D. melanogaster* as the template. BLAST (Tblastn) was used here. We have two reasons to perform additional annotation: 1) recently evolved genes are often poorly annotated; 2) annotation quality of outgroup species is presumably worse compared to *D. melanogaster* and we need to correct this bias. For particularly interesting cases (*e.g.* polycistronic coding genes), we searched literatures describing their evolutionary history.

*Conflicting cases could be classified into six categories*

We implemented a series of customized rules to call the presence of ortholog of gene *A* in species *B*. The first set of rules are used to call presence of ortholog based on gene prediction. For a synteny-predicted candidate orthologous region in *B*, we ran Tblastn to predict whether this region encodes an orthologous protein of *A*. If Tblastn could align the protein of *D. melanogaster* beyond the following thresholds (identity cutoff > 70% & coverage cutoff > 30%, identity cutoff > 30% & coverage cutoff > 70%, identity cutoff > 50% & coverage cutoff > 50%), we believed that the ortholog is present. If the alignment meets with the threshold (identity cutoff < 30% & coverage cutoff < 30%), the ortholog of *A* is absent in *B*. For all other cases, we called them as “boundary cases” if there is also no ortholog annotated by FlyBase and Ensembl. A total of 80 candidate new genes fall in this category including 65 cases in the list of Kondo *et al.* and 15 cases in our list (S2 Fig).

Secondly, for 275 out of 318 new genes dated by Kondo *et al* but not by us (S2 Fig), we identified orthologs as supported by at least two independent sources (FlyBase, Ensembl homolog, Ensembl gene family tree, and/or prediction). For example, in case of *FBgn0027589*, the ortholog is present across all 12 *Drosophila* species, which is supported by both Ensembl and FlyBase. The remaining 43 new genes are misidentified due to other types of problems (*e.g.* annotation problem due to polycistronic structure such as *tal-1A*/*tal-2A*/*tal-3A*). All these 318 genes are marked as “Dating problem in Kondo *et al*” (S2 Fig). In the opposite scenario, 101 new genes are only identified by us, which could be divided into four cases: 1) for 50 genes called as old genes in the K-dataset, their new gene calling were also supported by lack of one-to-one orthologs annotated by Ensembl or FlyBase; 2) for 5 genes called as old genes in the K-dataset, they are subject to complex evolutionary trajectories (pseudogenization of parental copies), such as *FBgn0032740* and *Cyp6t1*; 3) for 10 cases excluded by the K-dataset, we examined phylogenetic trees provided by Ensembl and confirmed that new gene are derived as suggested by longer branch length; 4) for 36 genes excluded in the K-dataset, FlyBase and Ensembl do not annotate orthologous genes in the outgroup species for most genes, which are also consistent with the lack of Tblastn hits.

Compared to the K-dataset, the false positives and false negatives are much fewer in the G-dataset (S1 Fig and S2 Fig). In the G-dataset, we misidentified only 19 new genes with 14 cases caused by double or triple losses in the outgroups (*e.g.* *CG2291*). In our pipeline, we first searched against closely related species and then went for remotely related species. If at least two independent losses are needed to explain the phylogenetic distribution of orthologs, we will assign a young age to gene *A* by following the maximum parsimony. The underlying assumption is: 1) the possibility of double or triple losses should be low; 2) the genomic alignment between *D. melanogaster* and remotely related species is less reliable compared to that between *D. melanogaster* and closely related species. Consistently, we only identified 14 cases with support by at least one additional source (FlyBase, Ensembl). The remaining 5 cases are caused by lack of sensitivity of UCSC genome alignment. Both FlyBase and Ensembl annotate orthologs in the outgroup species, but synteny does not cover the corresponding regions. In opposite, genome alignment built spurious alignment in remotely related species for 49 new genes identified by Kondo *et al.* However, our Tblastn search could not identify a protein at all. Thus, we referred them as false negatives. All these 68 genes are put into the third category entitled with “Dating problem in this work” (S2 Fig).

A fourth category (“Not applicable or difficult for dating”) consists of genes which are most resistant for dating due to their sequence features undesirable for new gene identification. 242 specific new genes claimed by Kondo *et al.* belongs to this category (S2 Fig). For 178 out of 242 genes, we found that synteny is in conflict for outgroup species sharing the same phylogenetic relationship relative to *D. melanogaster* (e.g. *D. simulans* and *D. sechellia*). These genes are generally located in repetitive regions (*e.g.* tandem amplification). It is thus likely that the orthologous regions may not be equally well assembled or be subject to species-specific gene conversion across these outgroup species. We thus excluded these genes. For example, we masked 19 out of 242 genes including 12 *Ste* genes, 5 Y-linked genes and 2 genes encoded by contigs but not anchored to five major chromosome arms. *Ste* is the X-linked tandem gene families each with redundant copies (S3A Fig). In the UCSC Net track, the most assembles can only reach level 2 of one-way syntenic mapping, rather than the adequate reciprocal syntenic mapping as level 1. The closely related species (*e.g.* *D. simulans*) also encodes multiple copies, but the corresponding region is not fully assembled and filled with lots of gaps and many of these copies even cannot be assigned to chromosomes (S3B Fig). The size contrast of assemblies between *D. sechellia* and *D. melanogaster* suggests that the region in *D. sechellia* might not be properly assembled due to its repetitive structure (S3A and S3C Fig).

In order to date each member correctly, high-quality outgroup genome must be available first. As for 5 Y-linked genes, Koerich *et al* assigned all of them to be old genes[11]. The remaining 45 genes consist of three subtypes: 1) 21 fast-evolving small proteins (<100 amino acids), since the protein sequence is too short and has a fast evolve speed, it is hard to determine the ages of these group genes; 2) 20 polycistronic genes without accurate previous literature support since the ortholog definitions are divergent: ages of those gene defined by different methods are inconsistent, but the outgroup species have synteny sequences and the predicted protein sequence identity or coverage is less than 50%. It is impossible to fully believe that it is a real protein and or not. Therefore, it is difficult to determine whether there are protein sequences in the outer group species, and the authentic gene age is also uncertain; 3) 4 tandem duplicates: it is difficult to distinguish from paralog: in the process of gene evolution, due to various reasons such as conversion, it is difficult to accurately determine the age of the tandem duplication gene. it is Different from the aforementioned 178 genes, the syntenic information is consistent across outgroup species suggesting that they are old genes. The small proteins or polycistronic genes are poorly annotated across outgroups. For 4 duplicates, Ensembl phylogenetic tree could not provide diagnostic information to infer the duplication order. Thus, we believed that all these three subtypes are difficult to date as of now. In our private new gene list, 45 candidates also belong to gene families. Similar to the above 4 tandem duplicates, the synteny is consistent across outgroup species showing that these 45 genes are derived copies in their respective families. However, Ensembl phylogenetic tree could not provide additional support. So, we also put these genes into the same fourth category.

The fifth category (“Different ortholog definition”) only consists of 28 private new genes identified by Kondo *et al* (S2 Fig)*.* For 24 out of 28 cases, the UCSC syntenic chain only covers a small portion (<30%) of coding regions or mainly corresponds to untranslated regions (UTRs) in outgroup species. Since the dating of Kondo *et al* is protein-centric, they called these genes as new genes. By contrast, our dating pipeline works on DNA-level and identified these genes as old genes. The reason why we took the age of most conserved exons to represent the age of whole genes is that these exons usually represent most important functional regions. Moreover, by performing dating on DNA-level, our dating does not depend on annotation quality of outgroup species. Actually, for all 24 cases, whether the coding region is accurately annotated is unknown due to the lack of protein evidence. For the remaining 4 cases, they all represent translocated genes. In our terminology, we only referred derived duplicate or orphan genes as new genes. By contrast, Kondo *et al.* interpreted incorrectly translocated genes as new genes.

The sixth and final category refers to the aforementioned 12 entries not comparable due to “Gene model change” (S2 Fig).

In the main text, we merged “Gene model change”, “boundary cases” and “different ortholog definition” as one dubious (in green color of S1B Fig) category to simplify.

*D. melanogaster-specific gene identification*

Candidate new genes were initially collected from previous studies [2,7,12]. We removed from this list of 233 candidates: 1) any genes whose *D. melanogaster*-specific release 6.05 (http://flybase.org) annotation status is 'withdrawn', 2) genes not located on the major chromosome arms 2L, 2R , 3L, 3R, or X, and 3) members of large tandem arrays, including the *Sperm dynein intermediate chain* [13,14], *Stellate* (*Ste*), and X: 19,900,000-19,960,000 arrays that are *D. melanogaster*-specific but are impossible to specifically study. We checked syntenic whole-genome alignments of the remaining 84 genes using our multi-species alignments at the UCSC Genome Browser. To be conservative, we required that all outgroups including the *D. simulans*, *D.sechellia*, *D. yakuba*, and *D. erecta* genome assemblies contained no assembly gaps, transposable elements, or repeats corresponding to the flanking regions of the putative *D. melanogaster*-specific gene. *D. melanogaster*-specific gene origination mechanisms and parental genes were taken from the original studies and confirmed using BLAT and BLASTp. If a gene had multiple significant (e < 10^-10^) full-length BLASTp hits *in D. melanogaster*, the hit that was most similar to the *D. melanogaster*-specific gene was assumed to be the parent. We used available *D. simulans* and *D. yakuba* next-generation sequencing reads to test the presence of putative *D. melanogaster*-specific tandem duplications in these two species[15,16]. We found no breakpoint spanning read pairs supporting *D. melanogaster* tandem duplications in any of 20 *D. simulans* or 20 *D. yakuba* genomes. Thus, these tandem duplications are specifically found in *D. melanogaster* and are not simply missing from the *D. yakuba* and *D. simulans* reference genome assemblies. We checked if any of the duplications in our final set are segregating rather than being fixed within *D. melanogaster* by analyzing 17 whole genome re-sequencing data from the DPGP2 core Rwanda (RG) genomes [17]. We required tandem duplications to have at least one read uniquely mapped to each of the three unique breakpoints in order to be called as 'present' in a particular line. Ten genes are not found in any of 17 additional *D. melanogaster* genomes we analyzed, suggesting that they are found specifically in the reference stock. Finally, we curated 10 *D. melanogaster-*specific genes. This dataset is actually a subset of G_K new gene data list.

**REFERENCES**

1. Kondo S, Vedanayagam J, Mohammed J, Eizadshenass S, Kan LJ, Pang N, et al. New genes often acquire male-specific functions but rarely become essential in Drosophila. Genes Dev. 2017;31(18):1841-6. doi: 10.1101/gad.303131.117

2. Zhang YE, Vibranovski MD, Krinsky BH, Long M. Age-dependent chromosomal distribution of male-biased genes in Drosophila. Genome Res. 2010;20(11):1526-33. doi: 10.1101/gr.107334.110. PubMed PMID: 20798392.

3. Shao Y, Chen C, Shen H, He BZ, Yu D, Jiang S, et al. GenTree, an integrated resource for analyzing the evolution and function of primate-specific coding genes. Genome Res. 2019;29(4):682-96. doi: 10.1101/gr.238733.118. PubMed PMID: 30862647; PubMed Central PMCID: PMC6442393.

4. Rhead B, Karolchik D, Kuhn RM, Hinrichs AS, Zweig AS, Fujita PA, et al. The UCSC Genome Browser database: update 2010. Nucleic Acids Res. 2010;38:D613-D9. doi: 10.1093/nar/gkp939. PubMed PMID: WOS:000276399100098.

5. Lynch M, Conery JS. The evolutionary fate and consequences of duplicate genes. Science. 2000;290(5494):1151-5. doi: 10.1126/science.290.5494.1151.

6. Rogers RL, Bedford T, Hartl DL. Formation and longevity of chimeric and duplicate genes in Drosophila melanogaster. Genetics. 2009;181(1):313-22. doi: 10.1534/genetics.108.091538.

7. Zhou Q, Zhang GJ, Zhang Y, Xu SY, Zhao RP, Zhan ZB, et al. On the origin of new genes in Drosophila. Genome Res. 2008;18:1446-55. doi: 10.1101/gr.076588.108.

8. Liebeskind BJ, McWhite CD, Marcotte EM. Towards consensus gene ages. Genome Biol Evol. 2016;8(6):1812-23. doi: 10.1093/gbe/evw113. PubMed PMID: 27259914; PubMed Central PMCID: PMCPMC4943184.

9. St Pierre SE, Ponting L, Stefancsik R, McQuilton P, FlyBase C. FlyBase 102--advanced approaches to interrogating FlyBase. Nucleic Acids Res. 2014;42(Database issue):D780-D8. doi: 10.1093/nar/gkt1092. PubMed PMID: 24234449; PubMed Central PMCID: PMCPMC3964969.

10. Kersey PJ, Allen JE, Armean I, Boddu S, Bolt BJ, Carvalho-Silva D, et al. Ensembl Genomes 2016: more genomes, more complexity. Nucleic Acids Res. 2015;44(D1):D574-D80. doi: 10.1093/nar/gkv1209.

11. Koerich LB, Wang XY, Clark AG, Carvalho AB. Low conservation of gene content in the Drosophila Y chromosome. Nature. 2008;456(7224):949-51. doi: 10.1038/nature07463. PubMed PMID: WOS:000261768300045.

12. Chen SD, Spletter M, Ni XC, White KP, Luo L, Long M. Frequent recent origination of brain genes shaped the evolution of foraging behavior in Drosophila. Cell Rep. 2012;1(2):118-32. doi: 10.1016/j.celrep.2011.12.010. PubMed PMID: 22832161; PubMed Central PMCID: PMCPMC4382513.

13. Nurminsky DI, Nurminskaya MV, De Aguiar D, Hartl DL. Selective sweep of a newly evolved sperm-specific gene in Drosophila. Nature. 1998;396(6711):572-5. doi: 10.1038/25126. PubMed PMID: 9859991.

14. Yeh SD, Do T, Chan C, Cordova A, Carranza F, Yamamoto EA, et al. Functional evidence that a recently evolved Drosophila sperm-specific gene boosts sperm competition. Proc Natl Acad Sci. 2012;109(6):2043-8. doi: 10.1073/pnas.1121327109.

15. Green EW, Fedele G, Giorgini F, Kyriacou CP. A Drosophila RNAi collection is subject to dominant phenotypic effects. Nat Methods. 2014;11(3):222. doi: 10.1038/nmeth.2856

16. Rogers RL, Cridland JM, Shao L, Hu TT, Andolfatto P, Thornton KR. Landscape of standing variation for tandem duplications in Drosophila yakuba and Drosophila simulans. Mol Biol Evol. 2014;31(7):1750-66. doi: 10.1093/molbev/msu124. PubMed PMID: 24710518; PubMed Central PMCID: PMC4069613.

17. Ni JQ, Markstein M, Binari R, Pfeiffer B, Liu LP, Villalta C, et al. Vector and parameters for targeted transgenic RNA interference in Drosophila melanogaster. Nat Methods. 2008;5(1):49-51. doi: 10.1038/nmeth1146. PubMed PMID: 18084299; PubMed Central PMCID: PMCPMC2290002.
